# Ultrafast Plasmonics Beyond the Perturbative Regime: Breaking the Electronic-Optical Dynamics Correspondence

**DOI:** 10.1021/acs.nanolett.1c04608

**Published:** 2022-03-28

**Authors:** Andrea Schirato, Giulia Crotti, Mychel Gonçalves Silva, Danielle Cristina Teles-Ferreira, Cristian Manzoni, Remo Proietti Zaccaria, Paolo Laporta, Ana Maria de Paula, Giulio Cerullo, Giuseppe Della Valle

**Affiliations:** † Dipartimento di Fisica, Politecnico di Milano, Piazza Leonardo da Vinci, 32, I-20133 Milano, Italy; ‡ Istituto Italiano di Tecnologia, via Morego 30, I-16163 Genova, Italy; § Departamento de Física, Universidade Federal de Minas Gerais, 31270-901 Belo Horizonte, MG Brazil; ∥ Instituto Federal de Minas Gerais, Campus Ouro Preto, Ouro Preto 35400-000, MG Brazil; ⊥ Istituto di Fotonica e Nanotecnologie - Consiglio Nazionale delle Ricerche, Piazza Leonardo da Vinci, 32, I-20133 Milano, Italy; # Cixi Institute of Biomedical Engineering, Ningbo Institute of Industrial Technology, Chinese Academy of Sciences, 1219 Zhongguan West Road, Ningbo 315201, China; ∇ Istituto Nazionale di Fisica Nucleare - Sezione di Milano, Via Celoria, 16, I-20133 Milano, Italy

**Keywords:** Plasmonics, Nanooptics, Hot Electrons, Ultrafast Spectroscopy, Ultrafast Nanophotonics

## Abstract

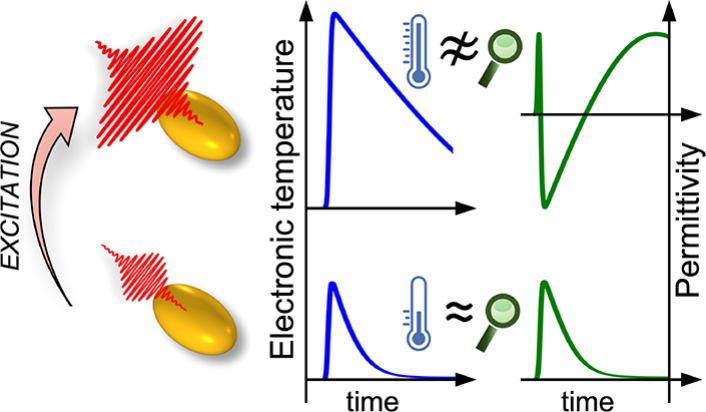

The transient optical response of plasmonic nanostructures has recently been the focus of extensive research. Accurate prediction of the ultrafast dynamics following excitation of hot electrons by ultrashort laser pulses is of major relevance in a variety of contexts from the study of light harvesting and photocatalytic processes to nonlinear nanophotonics and the all-optical modulation of light. So far, all studies have assumed the correspondence between the temporal evolution of the dynamic optical signal, retrieved by transient absorption spectroscopy, and that of the photoexcited hot electrons, described in terms of their temperature. Here, we show both theoretically and experimentally that this correspondence does not hold under a nonperturbative excitation regime. Our results indicate that the main mechanism responsible for the breaking of the correspondence between electronic and optical dynamics is universal in plasmonics, being dominated by the nonlinear smearing of the Fermi–Dirac occupation probability at high hot-electron temperatures.

Plasmonic nanomaterials are intensively investigated for a variety of applications,^1−3^ from innovative photodetection^4,5^ and photocatalysis^6−11^ to photothermal therapies^12,13^ and light harvesting.^14−16^ Moreover, optical components based on metallic nanostructures have lately emerged as a new promising platform for the manipulation of light^17−21^ with particular interest toward nonlinear nanophotonics applications.^22−25^

Underlying the advances envisaged above is the understanding of the nonequilibrium processes regulating the interaction between electromagnetic radiation and nanostructured plasmonic materials.^26−30^ In this framework, ultrafast optical spectroscopy has become the most suitable tool to gain insight into such processes (being noninvasive and of general applicability) and has given significant contributions to a vast literature (among the others, see, e.g., refs (31−36)), referred to as ultrafast plasmonics.^37,38^

In all of these studies, a fundamental hypothesis is the strict correspondence between the dynamics of the optical signal retrieved via the interaction with a low-intensity probe pulse interrogating the excited structure and the dynamics of the hot electron distribution (described by an electronic temperature) induced by absorption of the intense pump pulse. In particular, all studies so far assumed that the dynamics of the transient optical signal on the time scale of a few picoseconds after pump excitation correlates directly with the dynamics of the energy equilibration of hot electrons toward the metal lattice. However, to the best of our knowledge this hypothesis has not been systematically tested, and has even shown to be fulfilled in the presence of considerable effects altering the photoexcited carrier dynamics, such as ultrafast charge injection through metal–semiconductor heterojunctions.^39,40^ In this work, we aim at bridging this gap, relying on a combination of transient absorption spectroscopy, quasi-static electromagnetic modeling, and semiclassical theory of light–matter interaction to investigate the correspondence between electronic and optical dynamics in plasmonic nanostructures.

The optical properties of nanostructures are dictated by a complex interplay between the permittivity ε of the constituent material and their nanoscale geometry. Upon excitation with ultrashort light pulses, the latter can however be considered unvaried for a wide range of interaction conditions, and the transient optical response can be exclusively ascribed to the dynamical modification of material permittivity, *Δε*.^41^ As such, the nonperturbative regime of photoexcitation arises from two distinct mechanisms: (i) the nonlinear relationship, specific to the nanostructure geometrical configuration, between the nanostructure permittivity variation and the optical observable under consideration; and (ii) the optical nonlinearity detailing at the nanoscale the dependence of *Δε* on the excitation local intensity, depending on the material photophysical properties.

To illustrate these phenomena, we considered a prototypical plasmonic system represented by a gold nanoellipsoid embedded in a homogeneous medium. A typical transmission spectrum for such gold nanoparticles (NPs) with an aspect ratio of ∼2 dispersed in water computed from quasi-static formulas (Supporting Information, Section 1) is shown in Figure 1a (yellow curve). The extinction peak at low (high) photon energy can be ascribed to the excitation of the longitudinal (transverse) surface-plasmon resonance, LSPR (TSPR). Upon a change of the NP constituent medium permittivity *Δε*, the optical transmittance evolves from *T*(ε) to *T*(ε + *Δε*) . When *Δε* ≪ ε, a perturbative approach can be applied to determine *ΔT* = *T*(ε + *Δε*) – *T*(ε) = ∂*T*/∂ε × *Δε* (see, e.g., refs (42−44)). Yet, generally the evolution from *T*(ε) to *T*(ε + *Δε*) is not perturbative, and *ΔT* does not scale linearly with *Δε*, as depicted in Figure 1a. To quantify this effect, one can analyze spectra of the differential transmittance, *ΔT*/*T*, normalized to the corresponding *Δε*, and the effects induced by imaginary, *Δε*″, and real part, *Δε*′, of the permittivity change can be investigated separately. For the structure considered, the obtained two sets of spectra are reported in Figure 1b,c, for values of *Δε* (either imaginary or real) increasing from 0.01 to 2 (assumed constant over the entire spectrum for simplicity). The shape of these spectra can be interpreted as due to either the broadening (Figure 1b) or the red-shift (Figure 1c) of the LSPR/TSPR resonances, induced by the increase of material losses (*via Δε*″) and optical density (*via Δε*′), respectively. Importantly, since spectra are normalized to *Δε*, the onset of a nonperturbative effect manifests itself as the nonoverlapping of traces corresponding to different values of the *Δε*. This condition is more evident for the imaginary permittivity modulation (Figure 1b), compared to the real one (Figure 1c), and corresponds in any case to a relatively weak saturation effect even for a permittivity increase as high as 2, which is expected to give the strongest contribution to optical modulation around λ_1_ ∼ 500 nm (blue vertical line in Figure 1).

**Figure 1:**
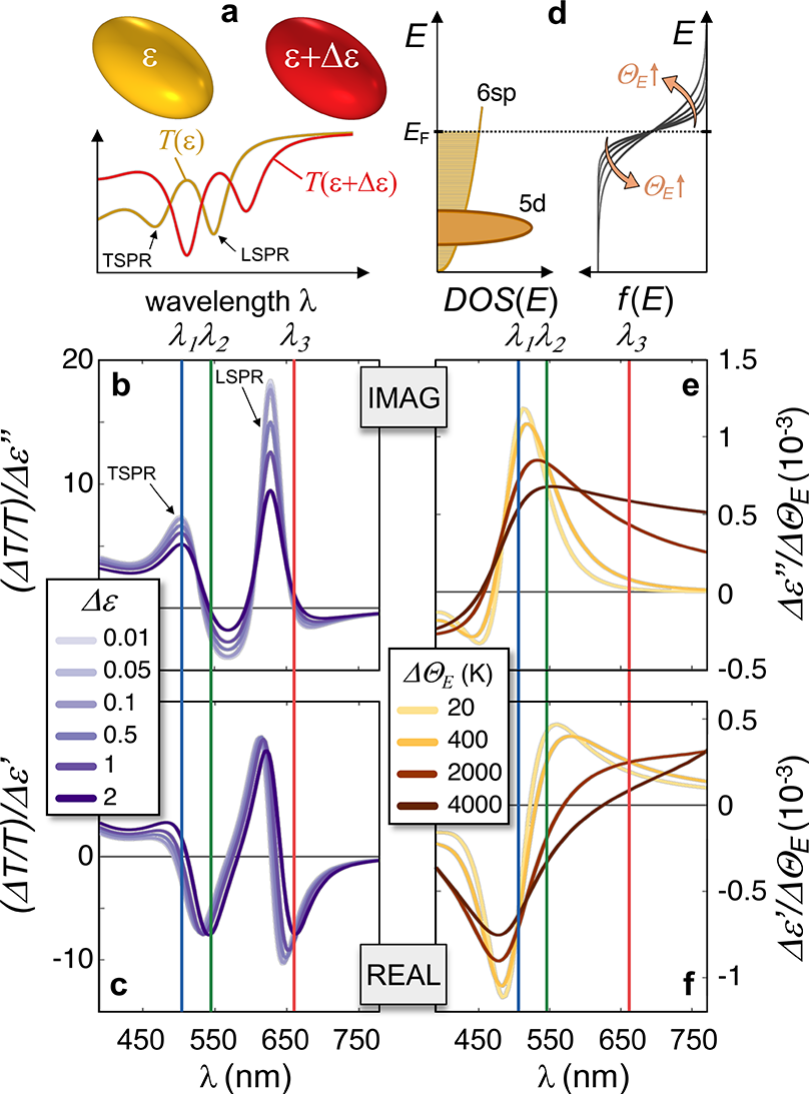
Nonperturbative photoexcitation of Au nanostructures. (a–c) Nonperturbative effect arising from the nonlinear relation between the system transmission *T* and the metal permittivity ε, for NPs in aqueous environment, as schematized in (a). The two plasmonic resonances (TSPR and LSPR) are marked by arrows. The differential transmittance signal, obtained for a given constant dispersionless *Δε* ranging between 0.01 and 2, is reported for either a purely imaginary (b) or real (c) variation. (d–f) Nonperturbative effect arising from the universal nonlinear mechanism of Fermi smearing in Au, triggered by the increase of the electronic temperature *ΔΘ*_E_ and resulting in a change of the metal electron Fermi–Dirac distribution *f*(*E*), as sketched in (d), where the arrows point from low to high temperatures. The *Δε* associated with the change in *f*(*E*), normalized to *ΔΘ*_E_, is displayed in both its imaginary (e) and real (f) parts. Vertical lines identify the three wavelengths analyzed in Figures 2 and 3

Furthermore, to account for the second aforementioned nonperturbative mechanism, we need to consider the dependence of *Δε* on the photoexcitation fluence. For noble metals, the most prominent effect following photoexcitation is the generation of hot carriers. These carriers, following an ultrafast electron–electron thermalization,^45^ are characterized by a Fermi–Dirac distribution in energy, , at temperature Θ_E_ higher than the room temperature Θ_0_, *E* being the electron energy and *E*_F_ the Fermi energy. The temperature increase *ΔΘ*_E_ = Θ_E_ – Θ_0_ causes a modification of *f*(*E*, Θ_E_) with respect to the equilibrium distribution, an effect referred to as “Fermi smearing”^46^ and schematically depicted in Figure 1d. In essence, the electronic temperature increase results in a reduction (increase) of the occupation probability of the electron states below (above) the Fermi energy *E*_F_. This promotes a variation of the imaginary part of the metal permittivity, accounting for the decreased (increased) absorption for 5d-6sp interband transitions (left panel, Figure 1d) involving final states above (below) *E*_F_. This is accompanied by a variation in the real part of the permittivity, due to the Kramers–Kronig relationship (Supporting Information, Section 2 for details). Because of the highly nonlinear dependence of *f*(*E*, Θ_E_) on the hot electron temperature Θ_E_ inherent in the Fermi–Dirac distribution, the Fermi smearing mechanism can significantly contribute to nonperturbative effects of the photoexcitation. A detailed illustration is provided in Figure 1e,f, showing respectively the spectra of the imaginary and real part of *Δε* due to thermalizd carriers only, normalized to the corresponding fixed *ΔΘ*_E_ considered in determining the interband transition modulation, here ranging from 20 to 4000 K (typical values induced by laser pulses with mJ/cm^2^ fluences). The fact that curves corresponding to different values of *ΔΘ*_E_ do not overlap represents a clear-cut indication of the onset of nonpertubative effects. A complex spectral behavior is also displayed in the two cases of real and imaginary permittivity modulations. In particular, a huge enhancement of *Δε*″ is predicted for λ > 550 nm (Figure 1e), whereas sign changes in the modulation of the real permittivity can take place over a relatively broad range of wavelengths from ∼520 to ∼620 nm (Figure 1f), in the red wing of the Au interband transition, starting at around 505 nm. Nonperturbative effects due to *Δε*′ should therefore govern the optical modulation at wavelengths as λ_2_ (green vertical line), while *Δε*″ is expected to dominate close to λ_3_ (red line). Note that the nonperturbative effects related to the Fermi smearing are intrinsic to any plasmonic system, that is, they do not depend on the specific resonances of the nanostructure, contrary to the nonperturbative mechanism previously introduced (Figure 1a–c) which is related instead to the connection between *Δε* and the nanostructure polarizability.

To reveal the occurrence of effects arising from the two phenomena discussed above, we performed nonperturbative transient absorption experiments to retrieve the normalized differential transmission *ΔT*/*T* for a sample of colloidal Au nanorods (NRs) dispersed in water (inset of Figure 2) with dimensions comparable to the one considered in the model in Figure 1. The capability of quasi-static formulas for nanoellipsoids to reproduce the static and transient optical response of small Au NRs has been ascertained in a previous work.^45^ The results of these measurements for four different fluences of the pump are reported in Figure 2a–c at three probe wavelengths, λ_1_ = 515 nm (Figure 2a), λ_2_ = 554 nm (Figure 2b), λ_3_ = 670 nm (Figure 2c), selected after having identified (in Figure 1) the spectral regions where nonperturbative effects are the strongest and dominated by the Fermi smearing. Full maps of the transient absorption signal are reported in Supporting Information, Section 4 (Figure S1).

**Figure 2:**
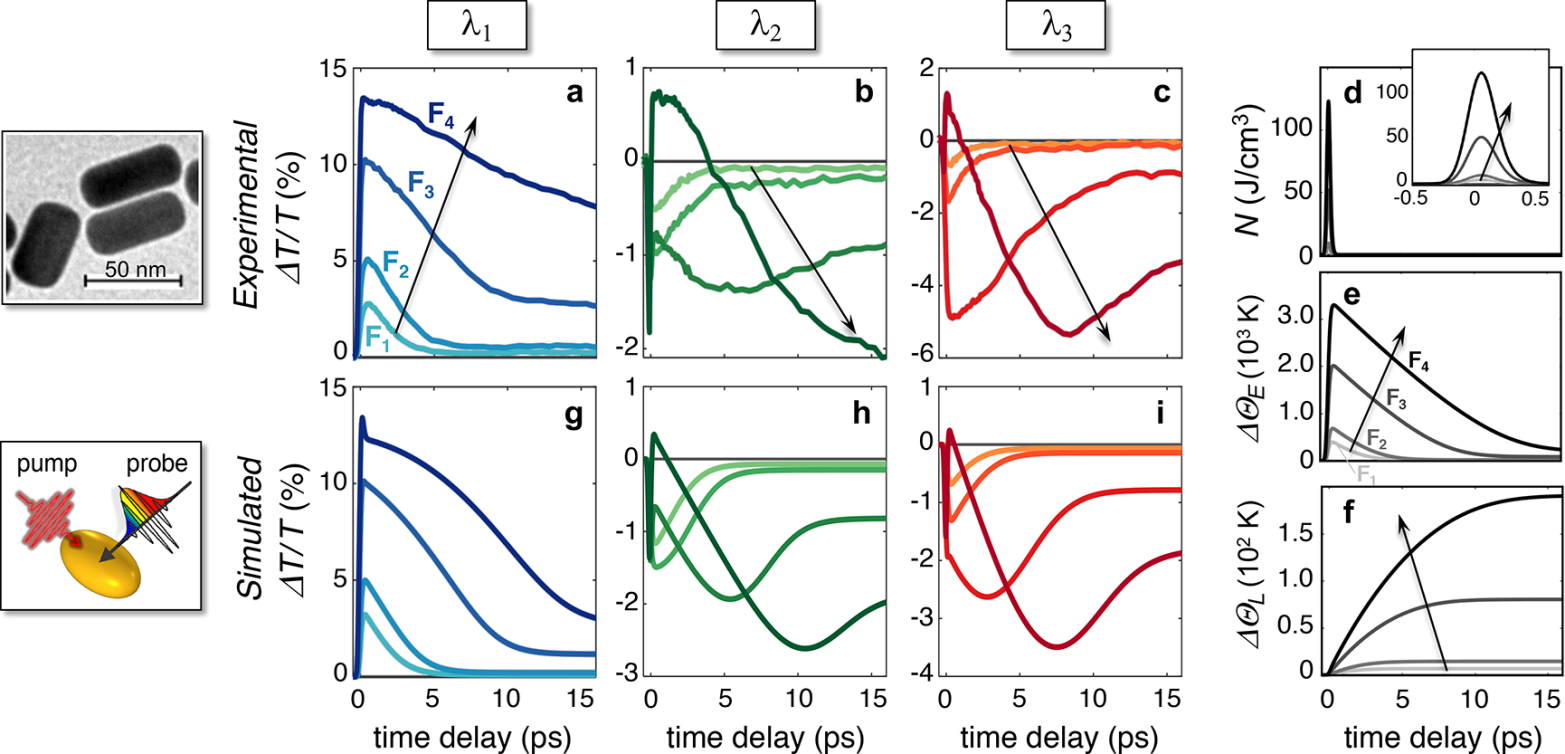
Breaking the correspondence between electronic and optical dynamics. (a–c) Experimental pump–probe traces recorded at three different probe wavelengths: (a) λ_1_ = 515 nm, (b) λ_2_ = 554 nm, and (c) λ_3_ = 670 nm. The sample is excited by laser pulses at 400 nm wavelength with 100 fs duration, whereas a broadband probe pulse is focused on the sample at a time delay *t* with respect to the pump. Further details, together with information on the experimental setup, can be found in the Supporting Information, Section 3. The color of the curves refer to different pump fluences with increasing fluence from lighter to darker shades. Pump fluences in the experiment are (in mJ/cm^2^), *F*_1_ = 0.13, *F*_2_ = 0.25, *F*_3_ = 1.26, and *F*_4_ = 3.12. (d–f) Simulated nonthermalized (d) and thermalized (e) electrons, together with lattice (f) dynamics under different excitation regimes. Inset in (d) is a magnification on the subpicosecond time scale. (g–i) Simulated *ΔT*/*T* signal evaluated at (g) λ_1_ = 505 nm, (h) λ_2_ = 546 nm, and (i) λ_3_ = 661 nm. Pump fluences in the simulations are (in mJ/cm^2^), *F*_1_ = 0.05, *F*_2_ = 0.11, *F*_3_ = 0.64, and *F*_4_ = 1.50. Insets show a transmission electron microscopy (TEM) image of the sample used in the measurements (top) and a schematic of the transient absorption scheme modeled in the simulations (bottom).

At λ_1_, nonperturbative effects manifest as a mere saturation of the *ΔT*/*T* with increasing pump fluence *F* (Figure 2a); apart from a slight modification in the signal dynamics, the *ΔT*/*T* remains monotonic with *F* with a peak reached in the first hundreds of femtoseconds and a slow decay on a picoseconds time scale. A strikingly different behavior is instead retrieved when analyzing the temporal evolution of the modulated signal at λ_2_ (Figure 2b). While traces corresponding to *F*_1_ and *F*_2_ follow, apart from the sign (depending on the spectral dispersion of the photoinduced *Δε*) the same dynamics as the curves at λ_1_ (cf. Figure 2a) a substantially different nontrivial evolution of the transient signal over time is observed for higher fluences. The signal starts rising (in absolute value) then decreases again within the first hundreds of femtoseconds. Because of this behavior, in the case of *F*_4_ the signal changes sign, reaching a second ultrafast peak after its initial negative-valued one. Following this abrupt sign reversal, the signal starts increasing (in absolute value) again, featuring a peak at ∼5 ps for *F*_3_ and at a time delay longer than 15 ps for *F*_4_. This is a remarkable delay effect considering that pump pulses have a duration of 100 fs. Also, the optical signal does not scale monotonically with fluence: by fixing a time delay, the highest modulated signal is not the one at the highest fluence, contrary to what happens at λ_1_. Such a nontrivial dynamics is not peculiar of λ_2_ only or restricted to a narrow band, since the same trend with fluence is also observed at a distant wavelength λ_3_ (Figure 2c). According to the full transient maps of Figure S1, the most pronounced nonperturbative effects are indeed observable in the red wings of the plasmonic resonances, which are more sensitive to photoinduced changes of the isosbestic lines (black contours in Figure S1). Note also that the onset of the nonperturbative regime is experimentally achieved for a pump fluence of *F*_3_ = 1.26 mJ/cm^2^, which is higher than typical low-perturbation fluences^26,32,47^ by a factor of ∼10 (at least) but still readily available and previously reported.^33,48^

To relate the observed optical dynamics to the temporal evolution of the hot carriers distribution, we simulated the transient absorption experiments using the so-called three-temperature model (3TM).^30,49−51^ It consists of a rate-equation model describing the energy relaxation processes following photoabsorption in terms of three internal energetic degrees of freedom of the nanostructure: *N*, the density of excess energy stored in a nonthermalized fraction of the out-of-equilibrium electronic population, the aforementioned temperature Θ_E_, accounting for the excitation level of thermalizd hot carriers, and Θ_L_, the Au lattice temperature (see Supporting Information, Section 5). For the plasmonic system under investigation, the temporal dynamics of these three internal variables are reported in Figure 2d–f for increasing values of the pump pulse fluence. As expected, *N* (Figure 2d), *ΔΘ*_E_ (Figure 2e), and *ΔΘ*_L_ (Figure 2f) monotonically increase with increasing fluence at any time delay *t*. To be more precise, *ΔΘ*_E_(*t*) scales proportionally although sublinearly with the pump fluence, which is a well-known consequence^41,47^ of the fact that the 3TM comprises a time-dependent coefficient, that is, the electronic heat capacity *C*_E_ ∝ Θ_E_(*t*).^52^ However, this affects the dynamics of *ΔΘ*_E_ only quantitatively, resulting in an increased time constant for the electronic temperature relaxation, which remains monotonic in time but tends to become linear for high fluences because of the increased electron thermal inertia. Note that the approximation of *C*_E_ as linearly dependent on Θ_E_ is justified by the predicted range of electronic temperatures we span (see Figure 2e). For higher fluences inducing temperatures exceeding ∼3000 K, more refined models^53^ should be employed to accurately assess the excited electron population dynamics. To retrieve then the transmission change of the ensemble of nanostructures via quasi-static formulas (see Supporting Information, Section 1), one should translate the dynamics of *N*, *ΔΘ*_E_, and *ΔΘ*_L_ into the corresponding permittivity modulation terms^25,30^ to compute spectra of *Δε* and *T*(ε + *Δε*) at each time delay (see Supporting Information, Sections 2 and 6).

The results of our calculations are reported in Figure 2g–i for three selected wavelengths: λ_1_ = 505 nm (Figure 2d), λ_2_ = 546 nm (Figure 2e), and λ_3_ = 661 nm (Figure 2f). Remarkably, by admitting a slight rigid shift (by less than 10 nm) and a lower value of fluence, ascribed to the increased Drude damping in Au static permittivity and to the linear model of pump absorption, neglecting saturation effects,^48^ simulations are in excellent agreement with measured data. As for experiments, the signal traces at λ_1_ (Figure 2g) have almost the same dynamics regardless of *F* apart from the increase in the decay time with the signal tail evolving from exponential to linear at high fluences. Conversely, the differential transmittance at λ_2_ (Figure 2h) and λ_3_ (Figure 2i) exhibits nontrivial abrupt changes in the first hundreds of femtoseconds, together with delayed peaks reached at several picoseconds, confirming the observed breaking of the electronic-optical dynamics correspondence for nonperturbative excitations: the dynamics of the optical signal (Figure 2a–c) cannot be directly employed, in general, to infer the dynamics of the hot carrier temperature (Figure 2e) upon high-fluence photoexcitation, creating an electron distribution at very high temperatures (Θ_E_ > ∼2000 K).

Our modeling approach allows us to gain further insight into the origin of the observed *ΔT*/*T* dynamics and in particular on whether the breaking of the electronic-optical correspondence is ascribable to a mere photonic effect (described in Figure 1a–c), is dominated by the Fermi smearing mechanism (detailed in Figure 1d,e), or rather both effects contribute.

We thus disentangled the contribution to the *ΔT*/*T* (for the fluence *F*_3_ = 640 μJ/cm^2^) arising from thermalizd carriers. This allows us to isolate the contribution of the electronic temperature, and rule out the possibility that the ultrafast dynamics of *ΔT*/*T* is due to nonthermal electrons^34^ (see Supporting Information, Section 7 for details). Moreover, to ascertain the role of Fermi smearing in the photoinduced modulation, the computations of the permittivity modulation, and of the ensuing differential transmittance, were performed starting from the same dynamics of Θ_E_ (solution of the 3TM) with the full nonperturbative as well as with a linearized model of variation in the hot electron distribution (see Supporting Information, Section 6). In the latter case, the variation of the Fermi–Dirac energy distribution of thermalizd hot carriers, written as *Δf*_T_(*E*) = *f*(*E*,Θ_E_) – *f*(*E*,Θ_0_) in the full model, is instead expressed as a linear function of *ΔΘ*_E_, that is, Δ*f*_T_(*E*) = [∂*f*(*E*, Θ_E_)/∂Θ_E_]_Θ_E_ = Θ_0_^*^_ΔΘ_E_ with Θ_0_^*^ an effective room temperature fitted to mimic broadening effects in Au interband transitions.^54^ With the same rationale as in Figure 1, the differential transmission is computed as if only the imaginary *Δε*″ (Figure 3c,h,m) or the real *Δε*′ (Figure 3e,j,o) components were changed by an increase in the electronic temperature *ΔΘ*_E_(*t*).

**Figure 3:**
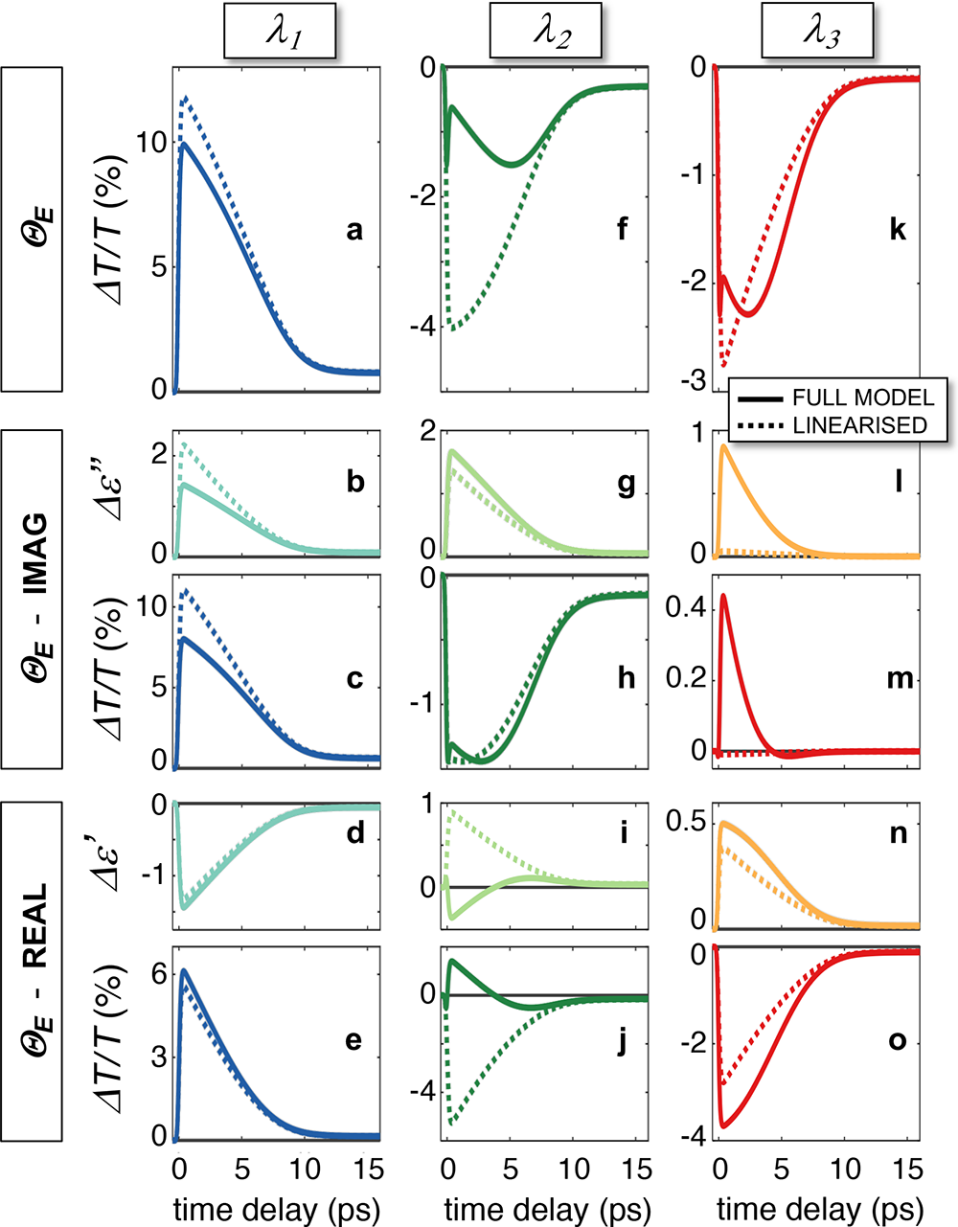
Disentangling contributions from thermalized hot carriers. (a) Simulated temporal dynamics of the pump–probe signal at λ_1_ = 505 nm due to thermalizd hot carriers only, namely computed as if *ΔΘ*_E_ was the only energetic variable modifying the metal permittivity. (b–e) Imaginary part of the photoinduced permittivity change from thermalized hot carriers (b) and corresponding contribution to the pump–probe signal trace at λ_1_, obtained as if *Δε*″ was the only term of permittivity modulation (c). With the same rationale, the real part *Δε*′ of permittivity modulation from Θ_E_ (d) and the resulting relative change in transmittance (e) are shown. (f–j) Same as (a–e) for quantities evaluated at λ_2_ = 546 nm. (k-o) Same as (a-e) at λ_3_=661 nm. Results from the full model, considering a non-perturbative occupation probability of thermal electrons (solid curves), and from its linearised version, considering a linear dependence on Θ_E_ of the thermal electron energy distribution (dashed curves) are compared

The main results of our analysis at λ_1_ are presented in Figure 3a–e for the two modeling approaches (solid lines for the full model, dashed lines for the linearized one), where the *ΔT*/*T* due to *ΔΘ*_E_ (Figure 3a) can be compared with the temporal evolution of the corresponding imaginary (real) part of permittivity variation *Δε*″ (*Δε*′), shown in Figure 3b (Figure 3d), and the relative transmission modulation, shown in Figure 3c (Figure 3e). As suggested by the regular trend of the pump–probe traces reported in Figure 2a,g, scaling monotonically with increasing fluence, the disentanglement confirms that the correspondence between optical (Figure 3a–e) and electronic (Figure 2e) dynamics at λ_1_ is preserved, the linearized model being adequate to describe the signal dynamics. At λ_2_ and λ_3_, first of all, as for the total *ΔT*/*T* (Figure 2b,c for experiments, Figure 2h,i for simulations), the signals arising from thermalized carriers are also similar (solid curves in Figure 3f,k) although the error introduced by the linearized Fermi smearing at λ_2_ is larger (compare dashed curves in Figure 3f,k). Most importantly, the onset of the delayed signal peak at around 3–4 ps, the fingerprint of the breaking of correspondence between optical and electronic dynamics, is lacking in the linearized model (dashed curves in Figure 3f,k). This indicates that such breaking is a signature of nonperturbative effects related to the Fermi smearing mechanism illustrated in Figure 1d. In more detail regarding λ_3_, Figure 3l,o show a dramatic discrepancy between the two models when dealing with imaginary permittivity modulations, where the linearized model largely underestimates the *ΔT*/*T* (cf. solid and dashed curves in Figure 3l,m). This correlates well with the analysis of Figure 1, since at this wavelength (marked by vertical red lines in Figures 1e,f) nonperturbative effects of the Fermi smearing, weak in terms of *Δε*′ (the curves in Figure 1f almost overlap), result in a sharp increase of *Δε*″ (Figure 1e). On the contrary, at λ_2_ (Figures 3g–j), the models mismatch is illustrated to be due to *Δε*′ (cf. solid and dashed curves in Figure 3i,j) and it is not only quantitative but involves a discrepancy in the sign of the contribution at the early stage of the dynamics. Again, this is fully consistent with the analysis reported in Figure 1, since at λ_2_ (marked by vertical green lines in Figure 1e,f) the Fermi smearing does not provide sizable nonperturbative effects due to *Δε*″ (the curves in Figure 1e are superimposed), whereas strong nonperturbative effects, including sign reversal, are predicted for *Δε*′ (Figure 1f) at high temperatures.

In conclusion, we investigated the ultrafast hot electron dynamics in plasmonic nanostructures excited with ultrashort laser pulses in a highly nonperturbative regime, reaching absorbed energy densities as high as ∼0.3 aJ/nm^3^ (yet well below the typical estimated^48^ damage threshold of a few aJ/nm^3^, as ascertained by the absence of morphological changes in the sample after pump irradiation). The theoretical predictions turned out to be in excellent agreement with transient absorption spectroscopy experiments performed on colloidal gold nanorods. Our model provides a consistent explanation of the origin of sign changes and unexpected formation of delayed peaks observed in the pump–probe traces despite the monotonic dynamics of hot electron relaxation taking place on the same time scale. These results indicate that the correspondence between electronic and optical dynamics ceases to be valid beyond the perturbative regime. This behavior is intrinsically related to the Fermi smearing mechanism presiding over hot electron relaxation in any metallic structure and is therefore universal in ultrafast plasmonics. In these terms, our study provides a fundamental argument with general validity on how the optical signal retrieved by ultrafast pump–probe spectroscopy relates to the electron temperature in metallic nanomaterials under high-fluence excitation. Moreover, our results will be relevant for understanding the nonequilibrium optical response of plasmon-enhanced nanophotonic devices, from ultrafast photodetectors to all-optical modulators, where the achievement of a nonperturbative regime of the optical excitation is of crucial relevance to address real-world applications.

## Supporting Information Available

Static optical response; permittivity modulation; experimental measurements; broadband transient absorption spectroscopy; the three-temperature model (3TM); modeling Au optical nonlinearities; nonthermal electron contribution (PDF)
