# Supplementary material for: Ultrafast Plasmonics Beyond the Perturbative Regime: Breaking the Electronic-Optical Dynamics Correspondence
Source: Nano Lett. 2022 Mar 28;22(7):2748–54. doi: 10.1021/acs.nanolett.1c04608 (PMC9011396; doi:10.1021/acs.nanolett.1c04608)
Supplement: Supplementary file 1 — nl1c04608_si_001.pdf [file nl1c04608_si_001.pdf]

Supporting Information for: Ultrafast Plasmonics Beyond the Perturbative Regime: Breaking the Electronic-Optical Dynamics Correspondence

Andrea Schirato,[†,‡] Giulia Crotti,[†,‡] Mychel Gonçalves Silva,[¶] Danielle Cristina Teles-Ferreira,[§] Cristian Manzoni,[‖] Remo Proietti Zaccaria,[‡,⊥] Paolo Laporta,[†,‖] Ana Maria de Paula,[¶] Giulio Cerullo,[†,‖] and Giuseppe Della Valle[*,†,‖]

†*Dipartimento di Fisica - Politecnico di Milano, Piazza Leonardo da Vinci, 32, I-20133 Milano, Italy*

‡*Istituto Italiano di Tecnologia, via Morego 30, I-16163, Genova, Italy*

¶*Departamento de Física, Universidade Federal de Minas Gerais, 31270-901 Belo Horizonte, MG, Brazil*

§*Instituto Federal de Minas Gerais, Campus Ouro Preto, Ouro Preto 35400-000, MG, Brazil*

‖*Istituto di Fotonica e Nanotecnologie - Consiglio Nazionale delle Ricerche, Piazza Leonardo da Vinci, 32, I-20133 Milano, Italy*

⊥*Cixi Institute of Biomedical Engineering, Ningbo Institute of Industrial Technology, Chinese Academy of Sciences, 1219 Zhongguan West Road, Ningbo 315201, China*

E-mail: giuseppe.dellavalle@polimi.it

1. Static Optical Response

To determine the optical response of an individual nanostructure upon plane wave illumination, its absorption, scattering and extinction cross-sections, σ_A, σ_S and σ_E respectively, should be defined. By considering a monochromatic linearly polarised plane wave in the quasi-static approximation (QSA), these quantities are readily expressed in terms of the particle polarizability, α, most generally a spectrally dispersed and complex-valued tensor.[1] For the specific case of nanoellipsoids, with wavelength dependent permittivity $\varepsilon(\lambda)$, embedded in a homogeneous medium with wavelength independent dielectric constant ε_m, the polarizability tensor $\alpha = \mathrm{diag}(\alpha_x, \alpha_y, \alpha_z)$ has diagonal components given by

$$\alpha_i(\lambda) = \varepsilon_0 V \frac{\varepsilon(\lambda) - \varepsilon_m}{L_i \varepsilon(\lambda) + (1 - L_i)\varepsilon_m}, \tag{S1}$$

where $i = x, y, z$, V is the nanoparticle volume, ε_0 the vacuum permittivity. The coefficients L_i are geometrical factors accounting for depolarization effects, reading:[1]

$$L_i = \frac{a_x a_y a_z}{2} \int_0^\infty f_i(q) dq, \tag{S2}$$

$$f_i(q) = \frac{1}{(a_i^2 + q)\sqrt{(a_x^2 + q)(a_y^2 + q)(a_z^2 + q)}}, \tag{S3}$$

for nanoellipsoids of semi-axes a_x, a_y, a_z. The absorption and scattering cross-sections are then computed as $\sigma_A = P_A/I_0$ and $\sigma_S = P_S/I_0$, with P_A and P_S the total power absorbed and scattered by the nano-object, respectively, and $I_0 = \frac{1}{2}\sqrt{\varepsilon_m} c \varepsilon_0 |\mathbf{E}_0|^2$ the incident intensity, given the incident (uniform) electric field $\mathbf{E}_0$. The total extinction cross-section is then simply given by the sum $\sigma_E = \sigma_A + \sigma_S$. In particular, regarding the absorbed power, P_A is defined as a volume integral of the ohmic losses power density over the nanoparticle. Under QSA, assuming that the incident electric field $\mathbf{E}_0$ induces a point-like electric dipole $\mathbf{p} = \varepsilon_m \alpha \cdot \mathbf{E}_0$, we have $P_A = \frac{\pi c}{\lambda} \mathrm{Im}\{\mathbf{p}^T \cdot \mathbf{E}_0\}$. On the other hand, the power scattered by the nanostructure can be expressed by introducing in the QSA leading order the effects of radiation reaction

onto the induced electric currents, which leads to $P_S = \frac{4\sqrt{\varepsilon_m}\pi^3 c}{3\varepsilon_0\lambda^4}|\mathbf{p}|^2$.

Note that, having an anisotropic polarizability (i.e. $\alpha_y = \alpha_z$ and $\alpha_x \neq \alpha_{y,z}$), spheroids with different orientation of the major axis with respect to the polarization of light exhibit different amplitudes of the two plasmonic oscillations. To mimic the random orientation of the nanorods (NRs) in the sample, the incident electric field in the simulations was written as $\mathbf{E}_0 = [\cos(\theta_x), \cos(\theta_y), \cos(\theta_z)]E_0$, with an average polarization angle $\theta_x = 75°$ to best fit the experimental absorbance (θ_y and θ_z being arbitrarily chosen, in view of the degeneracy $\alpha_y = \alpha_z$, under the constraint $\cos^2(\theta_x) + \cos^2(\theta_y) + \cos^2(\theta_z) = 1$).

Finally, when a realistic macroscopic sample is considered, e.g. an ensemble of single-size nanoellipsoids dispersed in aqueous solution with concentration N_p, the optical quantity of interest is the transmission of light, T, by the cuvette sample. Being L the cuvette thickness, Lambert-Beer's law gives

$$T = \exp\big[-\sigma_E(\alpha)N_pL\big]. \tag{S4}$$

For gold permittivity, a Drude-Lorentz model[2] fitted on experimental data[3] was adopted. Finally, in order to mimic the broadening of the plasmonic resonances caused by size inhomogeneities and scattering defects in the NR ensemble, an increased Drude damping Γ was introduced by following the same approach as the one reported in Ref. 4. We found a good agreement with the linear extinction measurements for $\Gamma/\Gamma_0 \simeq 2.5$, with $\Gamma_0 = 72$ meV the Drude damping in bulk gold.[2]

2. Permittivity Modulation

The temporal evolution of the electronic temperature Θ_E determines a variation of the occupation probability of thermalized electrons:[9,10]

$$\Delta f_T(E,t) = f\big[E, \Theta_E(t)\big] - f(E, \Theta_0) \tag{S5}$$

where $f(E, \Theta_E)$ is the Fermi-Dirac distribution and Θ_0 is the equilibrium temperature in static conditions. This modulation entails a modification ΔJ_T of the Joint Density of States (JDOS) for interband optical transitions near the L-point in the irreducible Brillouin zone, which, in turns, results in the variation of the absorption probability.[5,6] Precisely,

$$\Delta J_T(\lambda, t) = -\int_{E'}^{E''} D(E, \lambda)\Delta f_T(E, t)\mathrm{d}E, \tag{S6}$$

where $D(E, \lambda)$ is the energy distribution of the JDOS of the considered transition, with E the energy of the final state and λ the probe wavelength. Following the well-established[9] approach proposed by Rosei and co-workers,[5,6] we computed $D(E, \lambda)$ under parabolic band approximation and chose E' and E'', the effective masses, energy gaps and dipole matrix element as in the previously cited work.[6]

The following step consisted in deriving the variation of the imaginary part of the interband dielectric function at the probe wavelength λ:[7]

$$\Delta\varepsilon''_{\Theta_E}(\lambda, t) = \frac{e^2\lambda^2}{12\pi\varepsilon_0 m^2 c^2} |P_L|^2 \Delta J_T(\lambda, t) \tag{S7}$$

with m the electron mass an P_L the electric-dipole matrix element. Finally, Kramers-Kronig relations allow to retrieve the corresponding variation of the real part of the permittivity, $\Delta\varepsilon'_{\Theta_E}(\lambda, t)$.

Regarding the contribution to permittivity modulation arising from $N(t)$, the calculation of $\Delta\varepsilon_N(\lambda, t)$ proceeds similarly to $\Delta\varepsilon_{\Theta_E}(\lambda, t)$, the only difference being in the modulation of the electron occupation probability induced by N, which is not a Fermi-Dirac function, but rather a double step-like distribution.[8] $\Delta\varepsilon_{\Theta_L}(\lambda, t)$ is instead retrieved from the modification of the Drude permittivity, since a higher Au lattice temperature results in an increased Drude damping factor[12] and a reduced plasma frequency as a consequence of volume expansion.[9] The total modulation of gold permittivity is finally computed as the sum of the three contributions detailed above, *i.e.* $\Delta\varepsilon = \Delta\varepsilon_N + \Delta\varepsilon_{\Theta_E} + \Delta\varepsilon_{\Theta_L}$.

3. Experimental Measurements

The NR samples were synthesized by seedless growth methods[11] and characterized through UV-vis extinction measurements and transmission electron microscopy (TEM, more details in Ref. 10). The obtained NRs were in the form of prolate spheroids with aspect ratio $R \simeq 2$ (length $\simeq 50$ nm and diameter $\simeq 25$ nm) in water colloidal solution with molar concentration $n_p \simeq 0.6$ nM. Due to size inhomogeneities and scattering defects, the static response of the sample was found to be in satisfactory agreement with simulations results obtained for aspect ratio $R' = 2.4$ in the ideal monodispersed sample, and the correspondence between the two systems was set accordingly.

Transient absorption spectroscopy was performed with a regeneratively amplified Ti:sapphire laser (Libra, Coherent) with a fundamental at 800 nm with 1 kHz repetition rate, providing 100 fs pump pulses at the interband wavelength of 400 nm through second harmonic generation. The probe spectrum was obtained by white light continuum generation, by focusing the fundamental wavelength beam in a 3 mm sapphire crystal. A spectrometer (SP2150 Acton, Princeton Instruments) and a linear image sensor driven by a custom-built board (Stresing, Entwicklungsburo) allowed to measure the transmitted probe beam after the sample, with single-shot detection at the laser repetition rate.

4. Broadband Transient Absorption Spectroscopy

Transient absorption spectroscopy allows for determining the ultrafast dynamics of the photoexcited system over a broad spectral region. Specifically, Figure S1 displays the pump-probe signal over the visible range of wavelengths (from 450 to 700 nm) for a time delay up to 15 ps. Results of the experiments performed for the lowest (F_1) and highest (F_4) fluences (Fig. S1a and S1b, respectively) are compared to numerical simulations (Fig. S1c, S1d), showing a good agreement over the entire spectral region under consideration. The most pronounced mismatch is observed at the shortest wavelengths (highest energies), be-

tween 450 and $\sim$ 480 nm, where simulations partially fail in reproducing the dynamics of the negative lobe observed in the experiments.

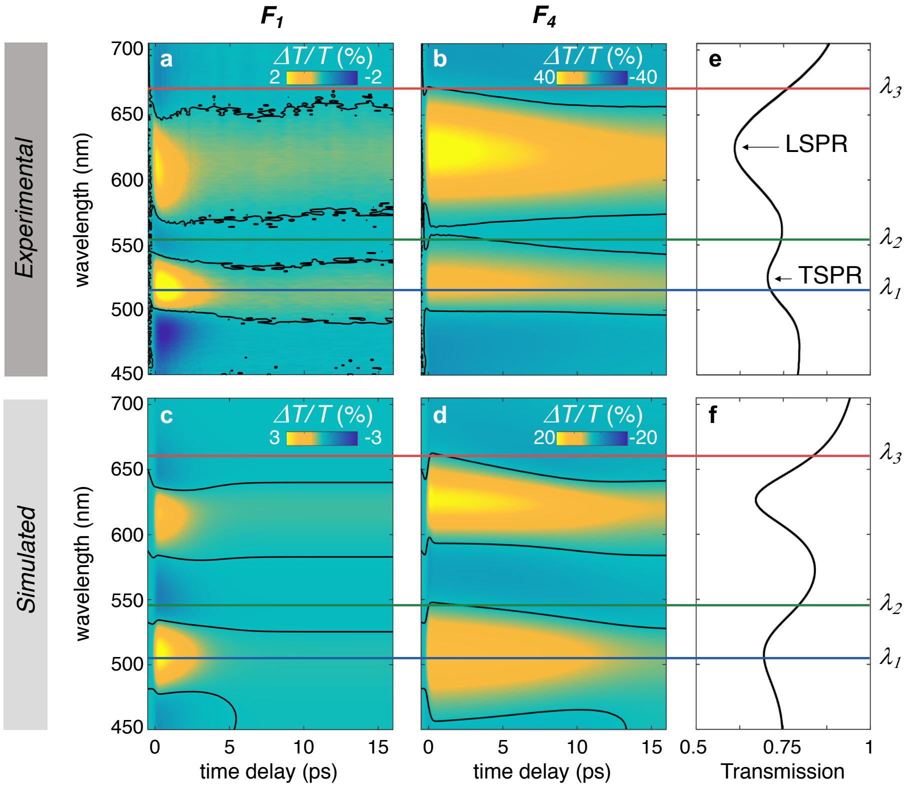

Figure S1: **Differential transmittance maps. a-d,** Experimental (a, b) and simulated (c, d) maps of the transient absorption spectroscopy signal for the lowest (F_1, 0.13 mJ/cm^2 for experiments. 0.05 mJ/cm^2 for simulations) and highest (F_4, 3.12 mJ/cm^2 for experiments, 1.50 mJ/cm^2 for simulations) fluences over a broad range of wavelengths. Black contours highlight the isosbestic points (*i.e. zeros of the* $\Delta T/T$ *signal).* **e-f,** *Experimental (e) and simulated (f) static transmission spectrum of the water colloidal solution with Au NRs. The two plasmonic resonances (TSPR and LSPR) are marked by arrows.*

We ascribe such a mismatch to the interband transitions contributing to the Au photoinduced permittivity modulation and not included in the model, which is accounting for the dominant one only (around the L point of the Brillouin zone, see Supporting Section 2). Moreover, Figure 1 shows the static transmission of the sample, for which again experiments (Fig. S1e) and simulations (Fig. S1f) are in good agreement in terms of intensity, spectral position and width of the two, longitudinal and transverse, resonances (marked by arrows) of the NR solution. Finally, across the maps of the differential transmittance signal, the three selected

wavelengths analysed in the main text are also highlighted (horizontal lines). Interestingly, the ones for which nonperturbative effects are discussed in the main text (λ_2 and λ_3) are shown to fall on the red wings of the LSPR and TSPR respectively, at the edges of the $\Delta T/T$ lobes delimited by the isosbestic points.

5. The Three-Temperature Model (3TM)

The three-temperature model (3TM)[8] reads as follow:

$$\frac{\mathrm{d}N(t)}{\mathrm{d}t} = -aN(t) - bN(t) + P_{\mathrm{abs}}(t), \tag{S8}$$

$$C_E \frac{\mathrm{d}\Theta_E(t)}{\mathrm{d}t} = -G\Big(\Theta_E(t) - \Theta_L(t)\Big) + aN(t), \tag{S9}$$

$$C_L \frac{\mathrm{d}\Theta_L(t)}{\mathrm{d}t} = G\Big(\Theta_E(t) - \Theta_L(t)\Big) + bN(t). \tag{S10}$$

Here, the coefficients a and b are coupling constants detailing the relaxation of non-thermalized electrons via electron-electron and electron-phonon scattering, respectively. $C_E = \gamma_E \Theta_E$ and C_L are the electron and lattice heat capacities, with γ_E the electron heat capacity constant, whereas G is the electron-phonon coupling coefficient. A detailed discussion on the estimation of all the parameters of the 3TM is reported in Ref. 9. Note that the linear dependence of C_E on Θ_E, as outlined in the main text, affects the electronic temperature dynamics in the nonperturbative regime. Indeed, such dependence results in an increased time constant for the hot electron temperature relaxation, which tends to become linear in time for very high fluences. The higher is the temperature, the higher is the heat capacity, *i.e.* the longer is the time required to exchange energy, because of the increased thermal inertia of the electronic population. Furthermore, $P_{\mathrm{abs}}(t)$ is the pump pulse absorbed power in unit of volume, representing the driving term modelling the ultrashort pump pulse photoexcitation

and expressed as a function of the pump fluence F by

$$P_{\text{abs}}(t) = \sqrt{\frac{2}{\pi}} \frac{\sigma_{\text{eff}}^{A}(\lambda_p) F}{V \tau_p} \exp\left(-2t^2/\tau_p^2\right). \tag{S11}$$

In this formula, σ_{eff}^{A} is the effective absorption cross-section at the pump wavelength ($\lambda_p = 400$ nm) and $\tau_p = 200$ fs the half width of the pump signal at $1/e^2$ power.

6. Modelling Au optical nonlinearities

In order to reproduce numerically the transient absorption spectroscopy measurements, we implemented a multi-step model for the photoexcitation and the subsequent energy relaxation processes taking place in plasmonic nanostructures upon illumination with ultrashort laser pulses. Figure S2 summarises our approach by illustrating a schematic of the model, starting from the information on the incident pump pulse (top left) to determine the dynamical $\Delta T/T$ signal (bottom right) to compare with experiments.

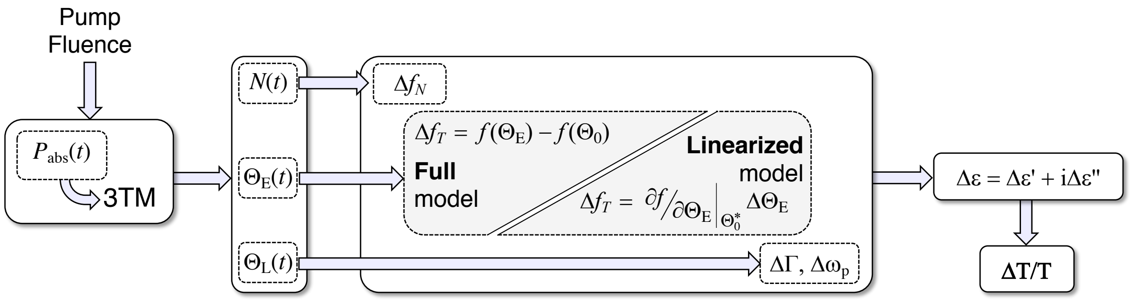

Figure S2: **Modelling Au optical nonlinearities upon ultrashort laser pulse illumination.** Schematic of the algorithm for the implementation of the numerical model employed to predict the dynamical evolution of the differential transmittance signal $\Delta T/T$ retrieved by transient absorption spectroscopy experiments.

The photoexcitation level of the structure is set by the fluence of the pump, its value being used to determine $P_{\text{abs}}(t)$, the power density (Eq. S11) absorbed by the nano-objects and following the time evolution of the pump pulse. Such quantity, as outlined in Supporting section 5, acts as the drive term of the 3TM, which in turn, when integrated, provides the

dynamics of the energetic degrees of freedom of the plasmonic nanostructure, *i.e.* $N(t), \Theta_E(t)$ and $\Theta_L(t)$. With those three time-dependent quantities at hand, the permittivity modulation of Au driven by each of them is then computed, following the procedure mentioned in the Supporting section 2. Specifically, (from top to bottom of the central panel in Fig. S2) the energy density stored in the nonthermal fraction of electrons $N(t)$ results in a modification of the electronic energy distribution Δf_N, the excited electron temperature Θ_E determines a change of the hot electron distribution Δf_T, and finally, as a consequence of an increased lattice temperature Θ_L, the Drude damping Γ and the plasma frequency ω_p are modified. Importantly, based on the same temporal evolution of the electronic temperature, two approaches can be pursued to determine the variation in the thermalised hot electron distribution Δf_T. According to the model we refer to as *full* in the main text, *i.e.* fully nonperturbative, Δf_T is at each time instant is given by the rigorous difference between the Fermi-Dirac distribution corresponding to an excited Θ_E and the static $f(E, \Theta_0)$, as written in Eq. S5. Conversely, when the linearised model is considered, such Δf_T is, indeed, linearised with respect to the electronic temperature variation. Its expression, written in the central box as well as in the main text, is given by the product of the temperature increase, $\Delta\Theta_E$, by an energy-dependent constant factor, computed as the partial derivative of $f(E, \Theta_E)$ with respect to Θ_E, evaluated at a given temperature Θ_0^*. As such, the linearised model treats Δf_T as if it has a constant distribution in energy (fixed by and corresponding to the one of such factor, depending exclusively on the electron energy), modulated in time by the dynamical evolution of the electronic temperature throughout the photoexcitation (the solution of the 3TM). As a result, no smearing of the Fermi-Dirac distribution is observed in the case of the linearised model, since the structure in energy of $f(E)$ is the same over time, regardless of the value of Θ_E. Subsequently, once the modification in the hot electron energy distribution has been computed for each time instant of the simulations, the model of Au thermo-modulational nonlinearities[5] outlined in Supporting section 2 is applied over time to determine the corresponding variation of Au permittivity (Eqs. S6-S7). Eventually,

corresponding to the modified time-dependent optical properties of the structure, an excited polarizability (Eq. S1) is then computed and employed to determine (by iteratively applying Eq. S4) the dynamical evolution of the system transmission and the differential signal $\Delta T/T$.

7. Nonthermal electron contribution

To ascertain the role of thermalised and nonthermal electrons as the origin of the non-trivial dynamics of the pump-probe signal experimentally observed and theoretically predicted, we employed our model to disentangle the contributions to the optical modulation arising from the three energetic degrees of freedom of the plasmonic nanostructure, that is, N, Θ_E and Θ_L. Figure S3 reports such disentanglement of the differential transmittance $\Delta T/T$ computed for the wavelength $\lambda_2 = 546$ nm examined in the main text, for the simulated fluences $F_1 = 0.05$ mJ/cm^2 (Fig. S3a-S3d) and $F_3 = 0.64$ mJ/cm^2 (Fig. S3e-S3h).

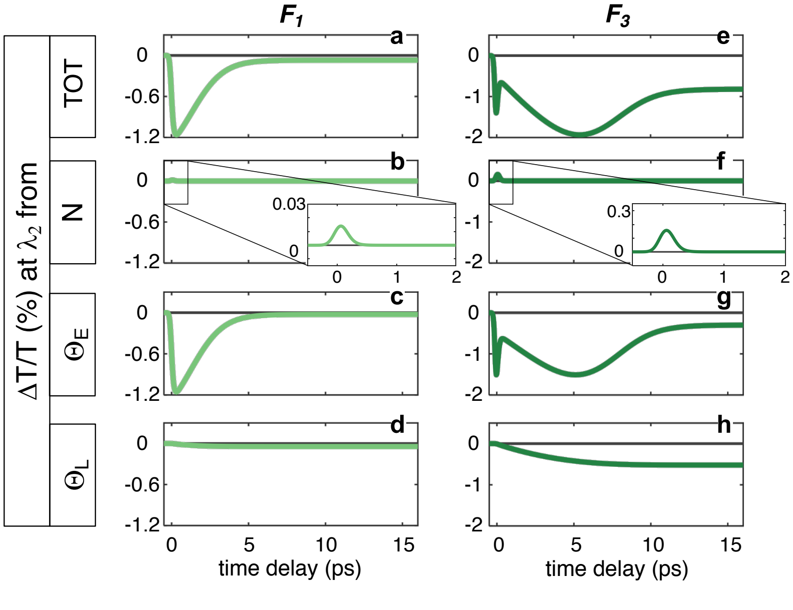

Figure S3: **Ultrafast differential transmittance signal disentanglement. a - d,** The total simulated $\Delta T/T$ signal (a) at $\lambda_2 = 546$ nm computed for a pump pulse fluence of $F_1 = 0.05$ mJ/cm^2 is disentangled in terms of contributions arising from nonthermal hot carriers (b), thermalised electrons (c) and the metal lattice (d).**e - h,** Same as (a - d) for a pump pulse fluence of $F_3 = 0.64$ mJ/cm^2.

Consistently with the discussion presented in the main text, the total signal (Fig. S3a,

S3e) is shown to be dominated for both fluences by the effect arising from thermalised electrons (Fig. S3c, S3g), which contribute to the optical modulation to a much larger extent than nonthermal carriers, mostly acting on an ultrafast (sub-ps) timescale (Fig. S3b, S3f) and the lattice, much slower (Fig. S3d, S3h). Moreover, also in the nonperturbative regime, thermalised hot carriers are demonstrated to be at the origin of the peculiar dynamics of the $\Delta T/T$. Indeed, by inspecting the dynamics of the disentangled signals at high fluence (right panels), the contribution arising from N (Fig. S3f) cannot explain the abrupt changes in the total differential transmittance (Fig. S3e), both for relative magnitude and sign (N gives a positive transmission modulation). On the contrary, the signal due to Θ_E (Fig. S3g, as well as Fig. 3f in the main text) precisely follows the non-trivial temporal trend of the total $\Delta T/T$, which can thus be understood by considering the non-perturbative effects discussed in the main text. Moreover, note that at low fluence, the $\Delta T/T$ due to thermalised electrons (Fig. S3c) follows the regular trend typical of the pump-probe signal in the weak perturbation regime (*i.e.* preserving the electronic-optical correspondence), and the negative peak observed at high fluence (Fig. S3g) disappears, since indeed due to non-perturbative nonlinear effects. Conversely, although scaled because of the decreased absorbed power, nonthermal electrons still give the same positive peak within the first ps as at high fluence (compare Fig. S3b and S3f).

In general, however, nonthermal and thermalised electrons can indeed produce a non-trivial interplay of contributions to the optical modulation, resulting in complex dynamics of the $\Delta T/T$ signal even in the weak perturbation regime, as they give effects which are dispersed in wavelength (possibly with opposite sign in certain spectral regions) and acting on delayed yet ultrafast timescales. To illustrate the phenomenon (accurately captured by our model, as also shown in previous reports[9,13,14]), we analysed the experimental optical modulation for the lowest pump fluence $F_1 = 0.13$ mJ/cm^2 at an exemplary wavelength λ_4 of 650 nm and compared measurements with simulations (performed with the linearised model for the hot electron energy distribution) at 635 nm for $F_1 = 0.05$ mJ/cm^2 (same

values as in the main text). Temporal traces of the differential transmittance are reported in Fig. S4a and S4b respectively. Indeed, an ultrafast non-trivial dynamics in the total $\Delta T/T$ is observed, with an ultrafast positive peak and a sign reversal within the first $\sim$ 300 fs, a negative peak reached at $\sim$ 500 fs, and a further sign change at $\sim$ 2 ps.

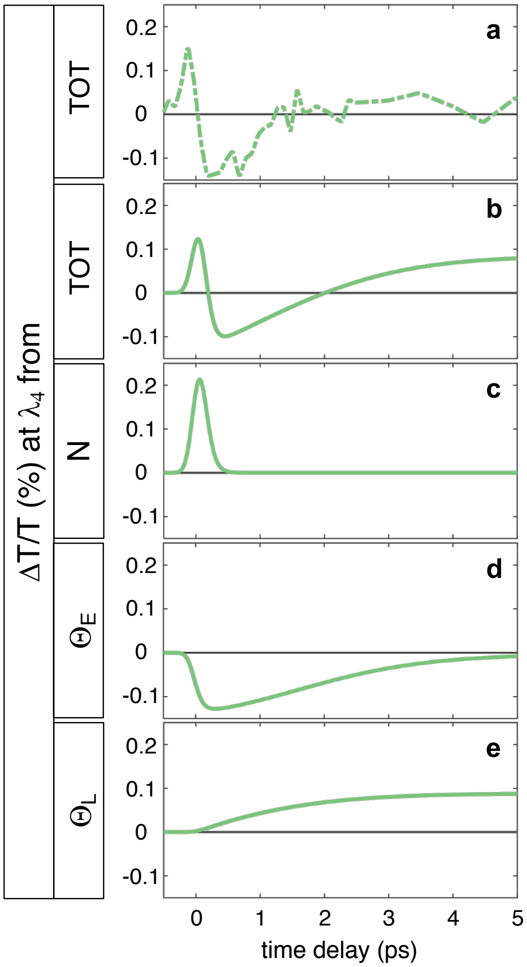

Figure S4: **Ultrafast dynamics of the differential transmittance signal. a,** Experimental pump-probe trace recorded at $\lambda_4 = 650$ nm for a pump fluence of $F_1 = 0.13$ mJ/cm^2. **b-e,** The total simulated $\Delta T/T$ signal (b) is disentangled in terms of contributions arising from nonthermal electrons (c), thermalised hot carriers (d) and the metal lattice (d). In the simulation, $\lambda_4 = 635$ nm and $F_1 = 0.13$ mJ/cm^2.

By disentangling the contributions to the $\Delta T/T$ arising from nonthermal electrons, thermalised carriers and the lattice, each of the main features of the dynamics can in fact be ascribed to one specific of the three terms. The first ultrafast peak could be interpreted as the fingerprint of nonthermal electrons, giving a positive contribution to modulation at 635

nm during the early times following photoexcitation (Fig. S4c). After hundreds of fs, the thermal electron contribution, negative at this wavelength, starts being the dominant one (Fig. S4d), explaining the change in sign at 300 fs. Then, at longer times, the contribution due to the lattice heating arises, bringing the signal from negative to positive values (Fig. S4e).

References

(1) S. A. Maier, *Plasmonics: Fundamentals and Applications*, Springer, New York (2007).

(2) Etchegoin, P. G.; Le Ru, E.; Meyer, M. An Analytic Model for the Optical Properties of Gold. *J. Chem. Phys.* **2006**, *125*, 164705.

(3) Johnson, P. B.; Christy, R.-W. Optical Constants of the Noble Metals. *Phys. Rev. B* **1972**, *6*, 4370.

(4) Husnik, M.; Linden, S.; Diehl, R.; Niegemann, J.; Busch, K.; Wegener, M. Quantitative Experimental Determination of Scattering and Absorption Cross-Section Spectra of Individual Optical Metallic Nanoantennas. *Phys. Rev. Lett.* **2012**, *109*, 233902.

(5) Rosei, R. Temperature Modulation of the Optical Transitions Involving the Fermi Surface in Ag: Theory. *Physical Review B* **1974**, *10*, 474.

(6) Guerrisi, M.; Rosei, R.; Winsemius, P. Splitting of the Interband Absorption Edge in Au: Temperature Dependence. *Phys. Rev. B* **1975**, *12*, 557.

(7) Marini, A.; Conforti, M.; Della Valle, G.; Lee, H.; Tran, T. X.; Chang, W.; Schmidt, M. A.; Longhi, S.; Russell, P. S.; Biancalana, F. Ultrafast Nonlinear Dynamics of Surface Plasmon Polaritons in Gold Nanowires due to the Intrinsic Nonlinearity of Metals. *New J. Phys.* **2013**, *15*, 013033.

(8) Sun, C.-K.; Vallée, F.; Acioli, L. H.; Ippen, E. P.; Fujimoto, J. G. Femtosecond-Tunable Measurement of Electron Thermalization in Gold. *Physical Review B* **1994**, *50*, 15337–15348.

(9) Zavelani-Rossi, M.; Polli, D.; Kochtcheev, S.; Baudrion, A.-L.; Béal, J.; Kumar, V.; Molotokaite, E.; Marangoni, M.; Longhi, S.; Cerullo, G.; Adam, P.-M.; Della Valle, G. Transient Optical Response of a Single Gold Nanoantenna: The Role of Plasmon Detuning. *ACS Photonics* **2015**, *2*, 521–529.

(10) Silva, M. G.; Teles-Ferreira, D. C.; Siman, L.; Chaves, C. R.; Ladeira, L. O.; Longhi, S.; Cerullo, G.; Manzoni, C.; de Paula, A. M.; Della Valle, G. Universal Saturation Behavior in the Transient Optical Response of Plasmonic Structures. *Physical Review B* **2018**, *98*, 115407.

(11) Wang, W.; Li, J.; Lan, S.; Rong, L.;Liu, Y.; Sheng, Y.;Zhang, H.; Yang, B. Seedless Synthesis of Gold Nanorods Using Resveratrol as a Reductant. *Nanotechnology* **2016**, *27*, 165601.

(12) Smith, J. B.; Ehrenreich, H. Frequency Dependence of the Optical Relaxation Time in Metals. *Phys. Rev. B* **1982**, *25*, 923.

(13) Della Valle, G.; Conforti, M.; Longhi, S.; Cerullo, G.; Brida, D. Real-Time Optical Mapping of the Dynamics of Nonthermal Electrons in Thin Gold Films. *Phys. Rev. B* **2012**, *15*, 155139.

(14) Dal Conte, S.; Conforti, M.; Petti, D.; Albisetti, E.; Longhi, S.; Bertacco, R.; De Angelis, C.; Cerullo, G.; Della Valle, G. Disentangling Electrons and Lattice Nonlinear Optical Response in Metal-Dielectric Bragg Filters, *Phys. Rev. B* **2014**, *89*, 125122.
